# Interpretable machine learning prediction of live birth after freeze-all FET cycles across transfer-order subgroups

**DOI:** 10.3389/fendo.2026.1868575

**Published:** 2026-07-17

**Authors:** Yu Zhao, He Wang, Lin Wang, Lei Yan, Jiao Liu, Mengyi Teng, Hao Wang, Ting Liu

**Affiliations:** 1 Reproductive Medicine Center, Dalian Women and Children’s Medical Group, Dalian, China; 2 Clinical laboratory, Second Affiliated Hospital of Dalian Medical University, Dalian, China; 3 School of statistics, Dongbei University of Finance and Economics, Dalian, China

**Keywords:** freeze-all FET cycles, interpretive nomogram, live birth, machine learning, SHAP, transfer-order heterogeneity

## Abstract

**Background:**

Transfer-order heterogeneity may affect live-birth prediction after freeze-all FET cycles, but existing prediction studies have rarely modeled first- and second-transfer records separately.

**Methods:**

We developed and compared logistic regression (LR), support vector machine, random forest, XGBoost, LightGBM, and CatBoost models in three related single-center analytical cohorts: an overall cohort comprising 990 eligible cycles, a first-transfer subgroup comprising 576 cycles, and a second retained transfer-record subgroup comprising 238 cycles. Feature templates denoted as T6, T8, and T10 refer to retained predictor sets containing 6, 8, and 10 variables, respectively. Model performance was evaluated using stratified 10-fold cross-validation, and binary metrics were summarized at receiver operating characteristic (ROC)-derived Youden thresholds.

**Results:**

CatBoost + T6 was retained for the overall cohort (area under the curve [AUC]=0.704) and the first-transfer subgroup (AUC = 0.812), whereas CatBoost + T10 was retained for the second retained transfer-record subgroup (AUC = 0.825). Parallel LR-based interpretive nomograms were used to support transparent presentation. Shapley additive explanations (SHAP) analysis showed recurrent contributions from female age and ovarian-reserve-related indicators, while the second retained transfer-record subgroup additionally highlighted basal progesterone and interval days.

**Conclusions:**

Transfer-order subgroup modeling improved model-population matching and provided more clinically interpretable structures for pre-transfer live-birth assessment after freeze-all FET cycles.

## Introduction

Infertility affects approximately one in six individuals worldwide, representing a substantial global health burden (1). Among contemporary assisted reproductive technologies, freeze-all FET cycles have become increasingly prominent (2, 3). The freeze-all strategy is partly supported by evidence that ovarian stimulation can impair endometrial receptivity in fresh-cycle transfer settings (4), and it can also reduce the risk of ovarian hyperstimulation syndrome when combined with appropriate trigger protocols (5). However, the strategy may prolong the treatment timeline and increase uncertainty during counseling. Accurate pre-transfer prediction of live birth may therefore help clinicians provide realistic outcome expectations and support individualized decision-making.

Previous studies conducted specifically in freeze-all cycles have identified several predictors of live birth. Ozgur et al. reported that female age, infertility duration, number of FETs, and blastocyst quality were significant predictors in 1,582 freeze-all cycles (6). Chen et al. identified endometrial thickness, age at oocyte retrieval, oocyte yield, prior IVF failures, infertility duration, and frozen embryo count as major predictors, but their reported C-statistic of 0.68 indicated only moderate discrimination (7). Wang et al. further examined cumulative live birth across multiple complete IVF cycles under a freeze-all strategy (8). These studies provide important evidence, but they generally analyzed mixed populations containing first and subsequent transfer cycles without explicitly modeling transfer order.

First and subsequent freeze-all FET records may differ systematically. Embryos available for later transfers have already undergone one selection process; endometrial preparation protocols may be adjusted after an earlier unsuccessful cycle; metabolic and endocrine status may change over time; and repeated treatment may affect patient expectations and treatment burden (9). Methodological research has also shown that pooling heterogeneous data can reduce model generalizability and subgroup calibration when between-group differences are not adequately addressed (10). Therefore, transfer-order stratification is not merely a descriptive subgroup analysis, but a clinically and methodologically justified modeling strategy.

Machine-learning models may capture nonlinear relationships and interactions that are difficult to represent with conventional regression alone. However, clinical use also requires transparent reporting, stable feature selection, calibration assessment, and interpretable model outputs (11–14). Accordingly, model development in this setting should balance predictive discrimination with clinically accessible explanation.

The present study aimed to develop and compare transfer-order-specific live-birth prediction models after freeze-all FET cycles. We analyzed an overall cohort, a first-transfer subgroup, and a second retained transfer-record subgroup under a harmonized feature-engineering framework; compared logistic regression (LR), support vector machine, random forest, XGBoost, LightGBM, and CatBoost; and used Shapley additive explanations (SHAP), rule-based interpretation, and LR-based interpretive nomograms to clarify subgroup-specific predictor structures. The clinical objective was to determine whether transfer-order subgroup modeling improves prediction, explanation, and pre-transfer counseling compared with pooled overall-cohort modeling.

## Methods

### Study design and participants

**Figure 1:**
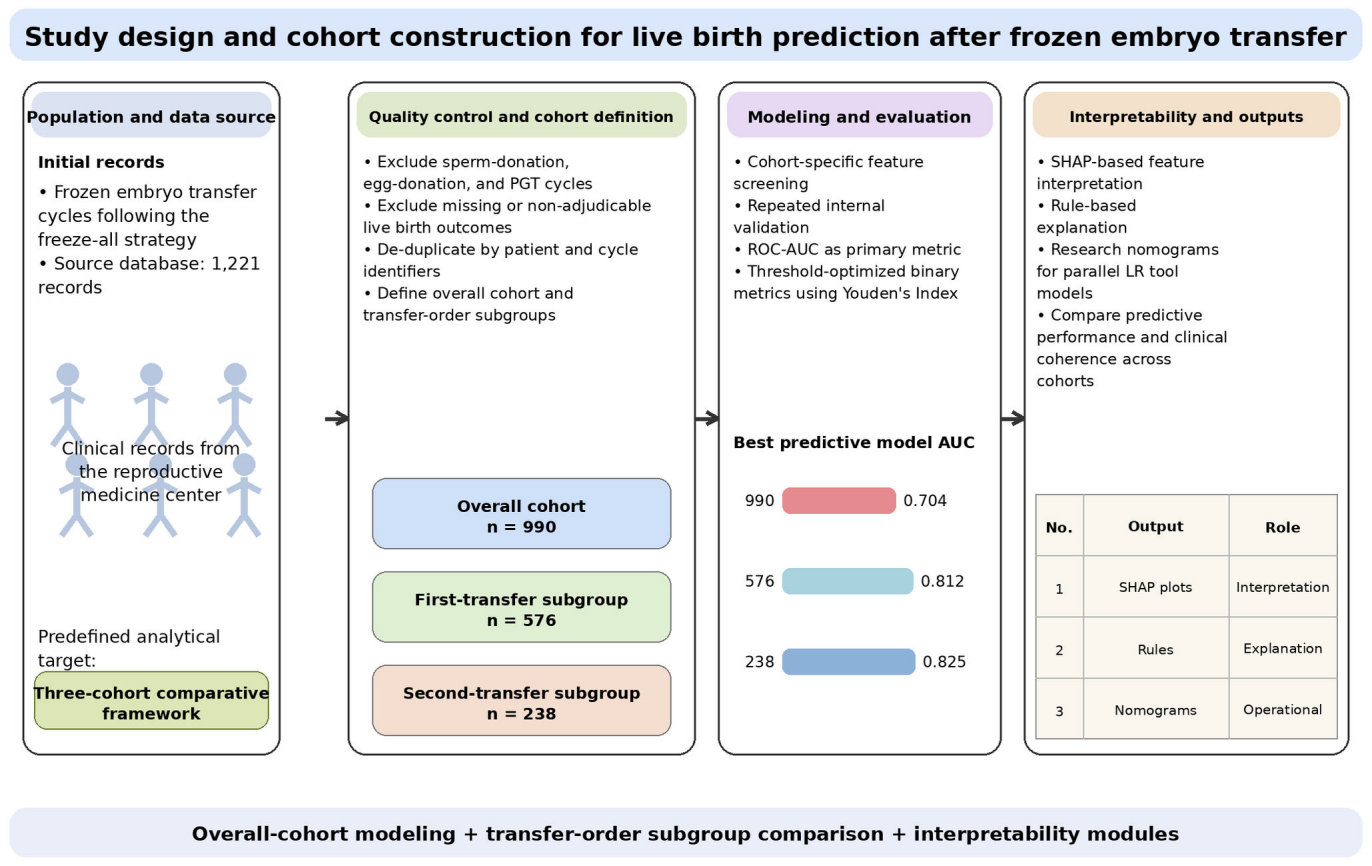
Study design, cohort construction, and analytical framework across the three cohorts.

This single-center retrospective prediction-modeling study used electronic medical record data from the Reproductive Medicine Center of Dalian Women and Children’s Medical Group. The analytical unit was an eligible FET cycle. Within each modeling cohort, only one eligible record per patient was retained for model development. The data collection period spanned March 4, 2021 to April 11, 2025. Frozen embryo transfer cycles following the freeze-all strategy were retrieved from the FET database. An initial pool of 1,221 records was obtained. Cycles involving sperm donation, egg donation, or preimplantation genetic testing were excluded. To ensure cohort consistency and outcome reliability, cycles with missing or non-adjudicable live-birth outcomes were excluded, duplicate records were removed using medical record number and cycle identifiers, and thaw-cycle records were retained according to transfer order. After quality control, three related analytical datasets were constructed from the same source database: an overall cohort comprising 990 first thaw-cycle records, a first-transfer subgroup comprising 576 patients with a single eligible retained FET record and a first thaw-cycle record, and a second-transfer subgroup comprising 238 later retained transfer records selected from patients with two eligible FET records. The second-transfer subgroup was therefore defined by transfer order in the retained database rather than exclusively by failure after the first transfer; in the available patient-level records, 237 of the 238 patients had no live birth after the first retained transfer record, whereas one patient had a prior live birth. These datasets were analyzed separately to examine transfer-order heterogeneity and could overlap with the overall cohort at the patient level. Because this was a prediction-modeling study rather than a causal comparison between balanced exposure groups, propensity-score matching or other baseline-balancing procedures were not applied. The overall cohort construction, subgroup definition, and downstream analytical framework are summarized in **Figure 1**.

### Ethics approval and consent to participate

This study was approved by the Ethics Committee of Dalian Women and Children’s Medical Group (protocol code FEJT-KY-2024-71, approval date: April 27, 2024).

### Variable definition and data preprocessing

Variable preprocessing followed the source-data workflow used to generate the three analytical cohorts. Starting from the 1,873-record raw source table, variables with more than 250 missing values were removed, three duplicated records were eliminated, and a small set of text-heavy variables was excluded before cohort construction. Embryo morphology was standardized and collapsed into four clinically merged categories (AA, AX, CG, and XX) before factor encoding to reduce sparsity. Numeric strings containing special symbols were cleaned under unified clinically consistent rules. Continuous variables were imputed with multivariate imputation by chained equations (MICE), and the categorical infertility-type variable was imputed by weighted random sampling according to the observed category distribution. Total AFC was calculated as the sum of follicles in both ovaries. The homeostatic model assessment of insulin resistance (HOMA-IR) was calculated as fasting insulin multiplied by fasting glucose and divided by 22.5. Encoded categorical predictors used in SHAP panels, retained feature templates, and interpretive nomograms represent factor-coded category labels rather than ordinal severity scores. According to the original data fields and preprocessing code, Infertility Type = 1, 2, and 3 corresponded to secondary infertility, other infertility type, and primary infertility, respectively. Medical Regimen = 1, 2, 3, 4, and 5 corresponded to GnRHa + HRT cycle, natural cycle, ovulation-induction cycle, ovarian-stimulation cycle, and hormone-replacement-therapy cycle, respectively. Embryo Morphology = 1, 2, 3, and 4 represented the AA-like morphology group, AX-like morphology group, compacting/cleavage-stage/rare morphology group, and non-AA/AX blastocyst morphology group, respectively. These encoded morphology variables should be interpreted as collapsed morphology groups rather than ranked embryo-quality scores. In the first-transfer subgroup, thaw cycle number is fixed at 1, so the retained Cycle No. terms in the LR nomogram are most appropriately interpreted as oocyte retrieval cycle numbers; in the second-transfer subgroup, Interval Days denotes the number of days between the first and second transfer dates.

### Feature selection and model development

To construct efficient and clinically interpretable live-birth prediction models, feature selection and model comparison were performed separately within each cohort under the same analytical framework. Candidate predictors were ranked using model-based feature-importance procedures, and compact feature templates were generated to support both prediction and interpretation. Template names indicate the number of retained predictors: T6, T8, and T10 refer to 6-, 8-, and 10-predictor feature sets, respectively. Multiple learners, including LR, support vector machine, random forest, XGBoost, LightGBM, and CatBoost, were evaluated within each cohort. The best predictive model was selected primarily for discrimination performance. When the best-performing model was not LR, a parallel LR tool model was retained for interpretive nomogram construction and cross-cohort comparison of transparent scoring structures. The LR tool model was therefore an interpretation layer rather than a replacement for the best nonlinear predictive model.

### Model evaluation and statistical analysis

Model performance was evaluated using stratified 10-fold cross-validation within each cohort to obtain stable estimates across resampling folds and to support robustness assessment. Because no patient contributed more than one record within any single modeling cohort, standard stratified 10-fold cross-validation was applied within each cohort rather than patient-grouped cross-validation. Discrimination was quantified primarily by the area under the receiver operating characteristic curve (ROC-AUC). For binary classification metrics, the decision threshold was not fixed at 0.5. Instead, the operating threshold was selected within each validation fold using Youden’s Index derived from the ROC curve, and threshold-optimized accuracy, precision, recall, and F1-score were then summarized across folds. This strategy was adopted because live-birth prediction represents an imbalanced medical classification setting in which a default threshold of 0.5 may be suboptimal. Comparative analyses across the 990 overall cohort, the 576 first-transfer subgroup, and the 238 second-transfer subgroup were interpreted descriptively to examine model-population mismatch and transfer-order heterogeneity. No external validation dataset was available for this single-center development study. All analyses were performed using Python and R with standard machine-learning libraries. For supplementary calibration assessment, Brier scores and calibration plots were calculated from the saved final test-split predicted probabilities of the final retained best predictive models. Because out-of-fold predicted probabilities from all cross-validation folds were not stored in the available modeling artifacts, this calibration analysis was interpreted as final-split supplementary evidence rather than as 10-fold out-of-fold calibration.

## Results

### Baseline characteristics across the three cohorts

Baseline characteristics were compared across the 990 overall cohort, the 576 first-transfer subgroup, and the 238 second-transfer subgroup in original clinical units before standardization. **Table 1** summarizes demographic, ovarian-reserve, and endocrine-metabolic variables available across all three analytical datasets. The comparison was descriptive and was not used for propensity-score matching or causal adjustment because the datasets were derived from the same source database and were not constructed as independent balanced groups. Female and male age, basal follicle-stimulating hormone (FSH), and basal luteinizing hormone (LH) showed statistically detectable between-cohort differences, whereas infertility duration, BMI, AMH, basal prolactin (PRL), basal estradiol (E2), basal testosterone (T), AFC, and HOMA-IR were broadly comparable.

**Table 1:** Baseline characteristics across the three analytical cohorts in original clinical units.

Variable	990 overall cohort (n=990)	576 first-transfer subgroup (n=576)	238 second-transfer subgroup (n=238)	P value
Female age (years)	34.00 (32.00-37.00)	34.00 (32.00-37.00)	35.00 (33.00-39.00)	0.002
Male age (years)	35.00 (33.00-39.00)	35.00 (32.75-39.00)	36.00 (34.00-39.75)	0.002
Infertility duration (years)	3.00 (2.00-5.00)	3.00 (2.00-5.00)	3.00 (2.00-5.00)	0.328
BMI (kg/m²)	22.58 (20.32-25.39)	22.31 (20.31-24.82)	22.86 (20.31-25.65)	0.469
AMH (ng/mL)	2.47 (1.17-4.19)	2.42 (1.12-4.30)	2.55 (1.29-4.33)	0.454
Basal FSH (IU/L)	6.42 (5.04-8.09)	6.41 (5.05-8.34)	5.88 (4.34-7.68)	0.003
Basal LH (IU/L)	4.64 (2.45-7.02)	4.71 (2.19-7.21)	4.05 (1.38-6.37)	0.008
Basal PRL (ng/mL)	15.73 (11.90-21.27)	15.59 (11.45-21.30)	15.91 (11.74-21.12)	0.784
Basal E2 (pg/mL)	41.60 (25.48-62.03)	39.45 (25.07-62.16)	39.84 (22.02-62.10)	0.932
Basal T (ng/mL)	0.26 (0.17-0.36)	0.26 (0.17-0.36)	0.26 (0.16-0.35)	0.649
AFC	13.00 (8.00-22.00)	13.00 (8.00-20.50)	14.00 (8.00-24.00)	0.677
HOMA-IR	2.18 (1.51-3.27)	2.18 (1.49-3.26)	2.09 (1.58-3.34)	0.816

Values are presented as median (interquartile range). Between-cohort comparisons were performed using the Kruskal-Wallis test and should be interpreted as descriptive comparisons rather than baseline adjustment procedures.

### Key predictors, cross-cohort performance, and interpretability synthesis

#### Overview of cohort-specific model evaluation

After describing cohort composition and subgroup structure, we evaluated model behavior within a harmonized screening framework and carried the comparison forward to the three final analytical cohorts. Formal ranking was based primarily on mean ROC-AUC under stratified 10-fold cross-validation, whereas accuracy, precision, recall, and F1-score were interpreted at ROC-derived Youden thresholds rather than at a fixed threshold of 0.5.

#### Global SHAP patterns across the final retained models

**Figure 2:**
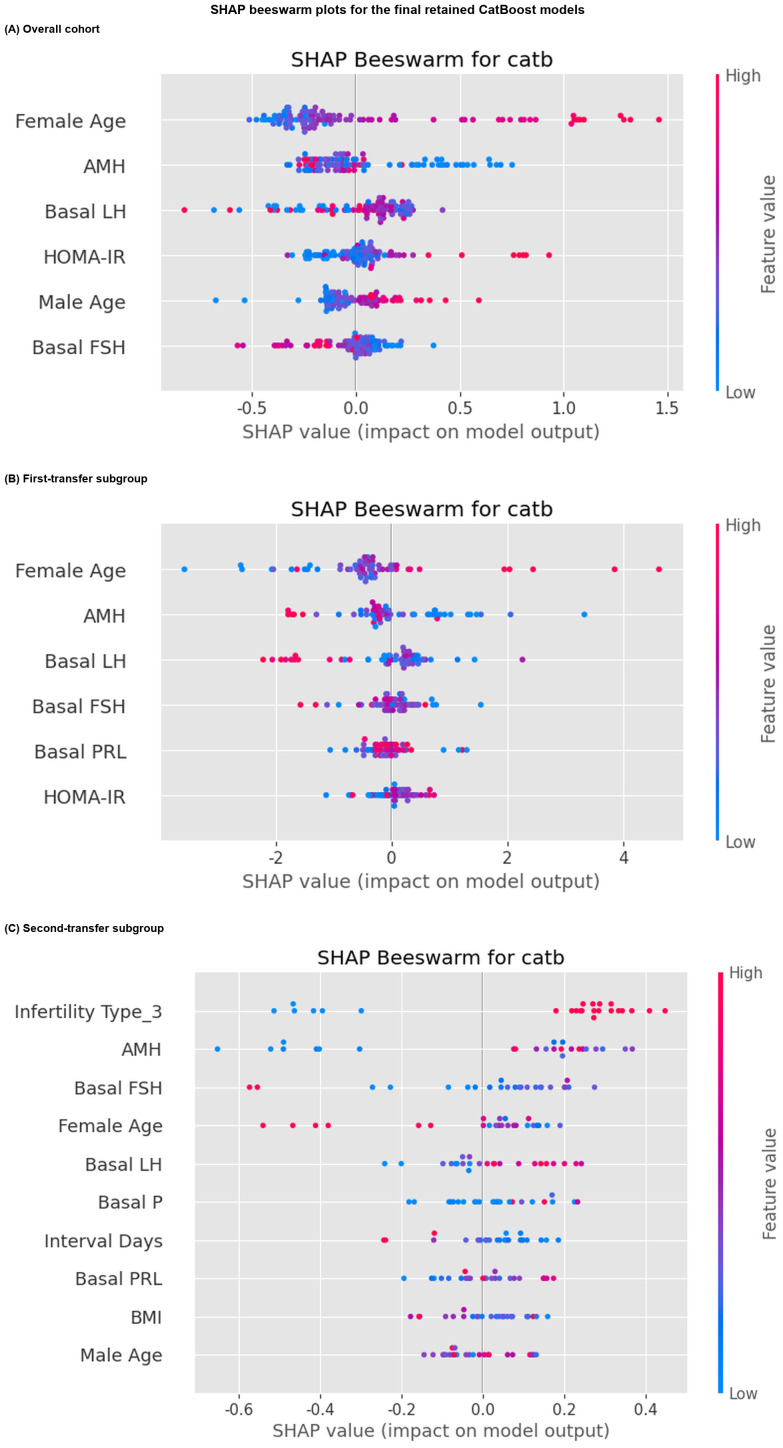
SHAP beeswarm plots of the best predictive models across the three cohorts: **(A)** overall cohort, **(B)** first-transfer subgroup, and **(C)** second-transfer subgroup. Encoded categorical labels denote factor-coded categories after clinically guided category collapsing; infertility type = 3 denotes primary infertility, and interval days denotes the number of days between the first and second transfer dates.

SHAP summaries for the final retained cohort-specific best models are shown in **Figure 2**. Positive SHAP values support live birth and negative values support non-live birth. Female age and ovarian-reserve-related indicators recurred across panels, whereas the second-transfer subgroup additionally showed stronger contributions from basal progesterone and Interval Days. In panel C, Infertility Type = 3 denotes primary infertility after factor coding, and Interval Days denotes the number of days between the first and second transfer dates.

#### Learner comparison and bridge to cohort-specific final models

We compared logistic regression, support vector machine, random forest, XGBoost, LightGBM, and CatBoost under the same analytical workflow and summarized their best screening-stage discrimination in **Supplementary Table 1**. Because the manuscript ultimately focuses on the three final analytical cohorts, the learner comparison is used here mainly as a bridge from harmonized screening to cohort-specific final model selection.

Nonlinear ensemble learners generally showed higher screening-stage discrimination than LR and SVM. The final retained best predictive models were CatBoost + T6 for the 990 overall cohort and the 576 first-transfer subgroup, and CatBoost + T10 for the 238 second-transfer subgroup. Parallel LR models were retained only for interpretive nomogram presentation and transparent comparison of scoring structures.

#### Cross-cohort explanation differences

Although the three cohorts shared several reproductive and endocrine signals, their explanation structures were not identical. The overall cohort captured mixed-population prognostic architecture, the first-transfer subgroup concentrated more strongly around ovarian reserve and basal hormone structure, and the second-transfer subgroup redistributed signal toward endocrine context, infertility type, and repeated-transfer process information. Together, SHAP, rule-based interpretation, and subgroup-specific tool models support subgroup-aware modeling rather than direct transfer of a model optimized in one subgroup to another.

**Figure 3:**
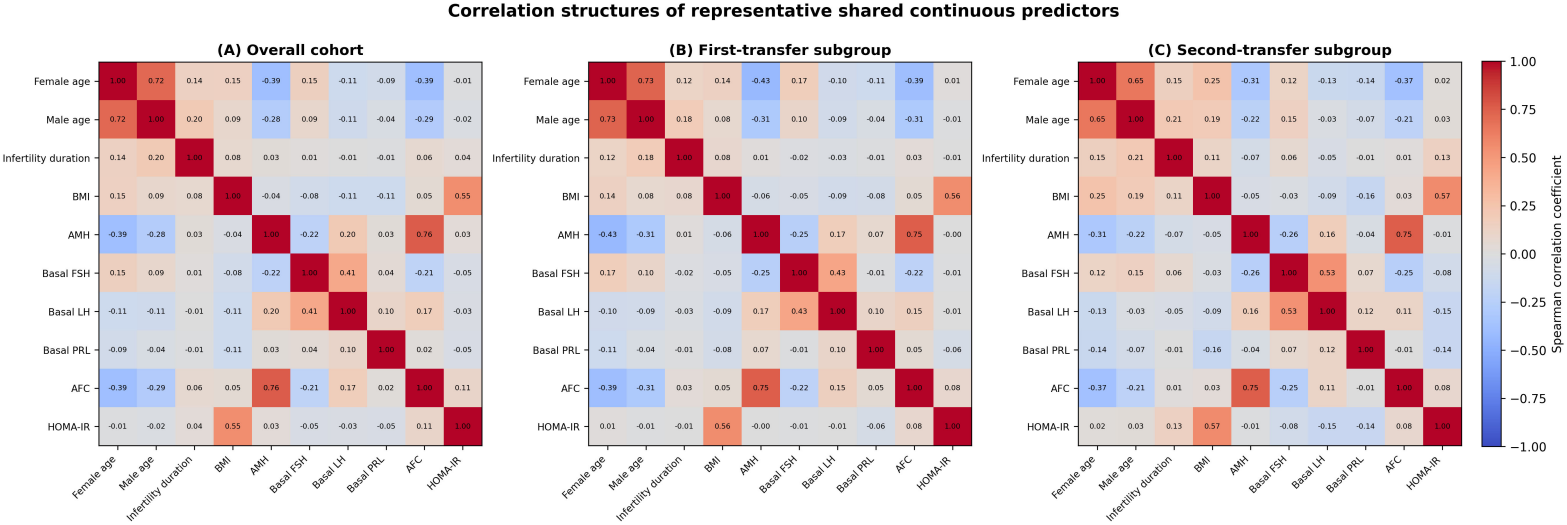
Correlation heatmaps of representative shared continuous predictors across the three cohorts: **(A)** overall cohort, **(B)** first-transfer subgroup, and **(C)** second-transfer subgroup.

The correlation structures of representative shared continuous predictors across the three cohorts are shown in **Figure 3**. Although the same broad biological domains recur in all three cohorts, the pairwise association patterns are not identical, further supporting the interpretation that transfer-order subgrouping changes not only performance but also predictor organization.

### Interpretation of cohort-level performance shifts

Cohort-level discrimination and threshold-optimized binary metrics are summarized in **Table 2**. Because binary metrics were calculated at ROC-derived Youden thresholds, they should not be interpreted as results obtained from a universal probability threshold of 0.5.

**Table 2:** Comparison of best predictive models and parallel LR tool models across three cohorts.

Cohort	Model role	Model	AUC	Accuracy	Sensitivity/Recall	Precision	F1	Youden threshold
990 Overall Cohort	Best predictive model	CatBoost + T6	0.704	0.678	0.637	0.775	0.691	0.633
990 Overall Cohort	Parallel LR tool model	LR + T8	0.680	0.649	0.576	0.773	0.640	0.631
576 First-Transfer Subgroup	Best predictive model	CatBoost + T6	0.812	0.750	0.867	0.545	0.657	0.109
576 First-Transfer Subgroup	Parallel LR tool model	LR + T10	0.770	0.770	0.732	0.631	0.650	0.211
238 Second-Transfer Subgroup	Best predictive model	CatBoost + T10	0.825	0.811	0.797	0.876	0.825	0.649
238 Second-Transfer Subgroup	Parallel LR tool model	LR + T8	0.737	0.773	0.890	0.770	0.815	0.530

T6, T8, and T10 denote retained feature templates containing 6, 8, and 10 predictors, respectively. LR denotes logistic regression. Sensitivity/recall, precision, F1, and threshold were calculated at ROC-derived Youden thresholds.

### Cross-validated robustness of the final retained models

To examine robustness beyond one selected configuration, we summarized the performance distribution of optimized LightGBM models under the same 10-fold cross-validation framework (**Supplementary Table 2**). These summaries indicate that optimization improved discrimination while preserving the cohort-specific ranking pattern of the final retained models.

**Figure 4:**
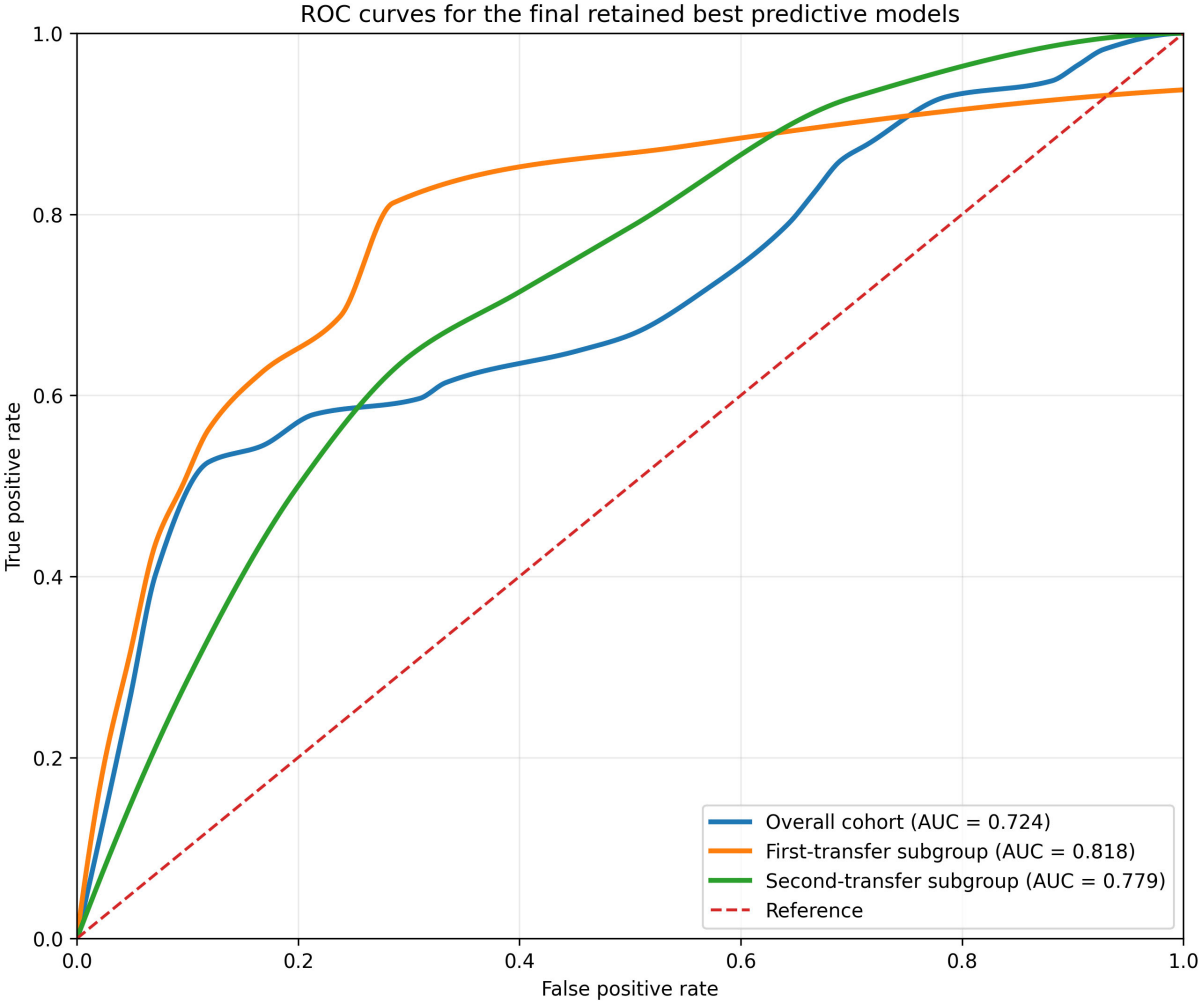
Smoothed ROC curves of the final retained best predictive models in the three cohorts. The curves provide a visual comparison based on saved model-prediction outputs, and the AUC labels correspond to the 10-fold cross-validated summary estimates reported in **Table 2**.

To provide a direct visual comparison of cohort-level discrimination, **Figure 4** shows smoothed ROC curves of the final retained best predictive models for the 990 overall cohort, the 576 first-transfer subgroup, and the 238 second-transfer subgroup. The formal performance comparison remains based on the 10-fold cross-validated summary estimates reported in **Table 2**, whereas the ROC curves provide a visual summary based on the saved model-prediction outputs.

#### Calibration assessment of the final retained models

Using the saved final test-split predicted probabilities, supplementary calibration assessment was performed for the final retained best predictive models. The Brier scores were 0.207 for the 990 overall cohort, 0.156 for the 576 first-transfer subgroup, and 0.196 for the 238 second-transfer subgroup. The corresponding expected calibration errors based on five quantile bins were 0.129, 0.081, and 0.106, respectively. These findings are provided as supplementary final-split calibration evidence in **Supplementary Figure 1** and **Supplementary Table 4**, while the primary model comparison remains based on the stratified 10-fold cross-validated discrimination and threshold-optimized metrics reported in **Table 2**.

### Interpretive rule and nomogram analysis across the three cohorts

#### Analytical framework

This section compares the 990 overall cohort, the 576 first-transfer subgroup, and the 238 second-transfer subgroup across three linked interpretive layers: co-occurrence patterns observed in the data, predictor structures used by the final predictive models, and LR-based interpretive nomogram expressions. This organization was used to make the subgroup-specific model outputs more accessible to clinical readers.

The 990 overall cohort and the 576 first-transfer subgroup included classical association rules, model interpretation rules, and interpretive nomograms. For the 238 second-transfer subgroup, the available interpretive materials emphasized the main predictive model, the supplementary simplified interpretation model, and the parallel LR tool model. All internal labels, figure titles, and variable labels were presented in English to avoid rendering inconsistencies in the final graphics.

When the final retained feature templates are compared directly across cohorts, a clear redistribution chain emerges rather than a simple increase or decrease in model complexity. The 990 overall cohort retained a compact 6-indicator best predictive template centered on AMH, Basal LH, Male Age, Female Age, HOMA-IR, and Basal FSH, together with an 8-indicator parallel LR tool template composed of Embryo Morphology category 4 (non-AA/AX blastocyst morphology group), Embryo Morphology category 3 (compacting/cleavage-stage/rare morphology group), Number of embryos transferred = 2, Medical Regimen category 4 (ovarian-stimulation cycle), Female Age, HOMA-IR, Male Age, and Basal T. This pattern indicates that in the mixed cohort the dominant information is still organized around a broad reserve-age-metabolic axis, whereas embryo and regimen variables are retained mainly in the operational explanation layer rather than in the best nonlinear predictive core.

The 576 first-transfer subgroup also retained a 6-indicator best predictive template, but its content contracted toward AMH, Basal FSH, Female Age, Basal LH, HOMA-IR, and Basal PRL. This contraction is clinically meaningful: once repeated-transfer history is removed, the prediction problem becomes more tightly aligned with ovarian reserve, female age, and basal endocrine responsiveness. This interpretation is consistent with the corresponding SHAP panel, in which Female Age, AMH, Basal LH, Basal FSH, Basal PRL, and HOMA-IR remain the dominant contribution axes, even though the exact SHAP order is not strictly identical to the retained-template listing. In this setting, Basal FSH and Basal PRL sharpen the reserve-hormone axis, whereas oocyte retrieval cycle number, encoded embryo morphology categories, Total AFC, number of embryos transferred, and medical regimen categories move into the 10-indicator LR tool template as refinement features that help translate a relatively concentrated biological signal into bedside scoring and communication. Thus, the first-transfer subgroup is not a reduced copy of the 990 cohort; rather, it is a more homogeneous setting in which the main determinant pathway is compressed into a narrower and more clinically streamlined template.

In contrast, the 238 second-transfer subgroup retained a 10-indicator best predictive template composed of AMH, basal progesterone (Basal P), primary infertility (Infertility Type = 3), Female Age, Basal FSH, Interval Days, BMI, Male Age, Basal PRL, and Basal LH. Here, Interval Days denotes the number of days between the first and second transfer dates. The expansion of this template is also clinically informative rather than merely technical. After a prior transfer, live-birth prediction is no longer determined only by reserve and age; it additionally depends on endocrine timing, inter-cycle recovery, infertility phenotype, metabolic background, and repeated-treatment history. The corresponding SHAP panel is concordant with this expanded structure, highlighting primary infertility (Infertility Type = 3), AMH, Basal FSH, Female Age, Basal LH, Basal P, Interval Days, Basal PRL, BMI, and Male Age as the main contribution axes. Basal P and Interval Days are especially important because they capture timing-related and process-related information that is largely absent from first-transfer prediction, whereas BMI and primary infertility reflect broader host-condition and etiologic constraints. Accordingly, although a simplified 4-indicator version may still be useful for exploratory operational screening, the present results support retaining the richer 10-indicator template as the principal analytical framework for the second-transfer subgroup.

**Figure 5:**
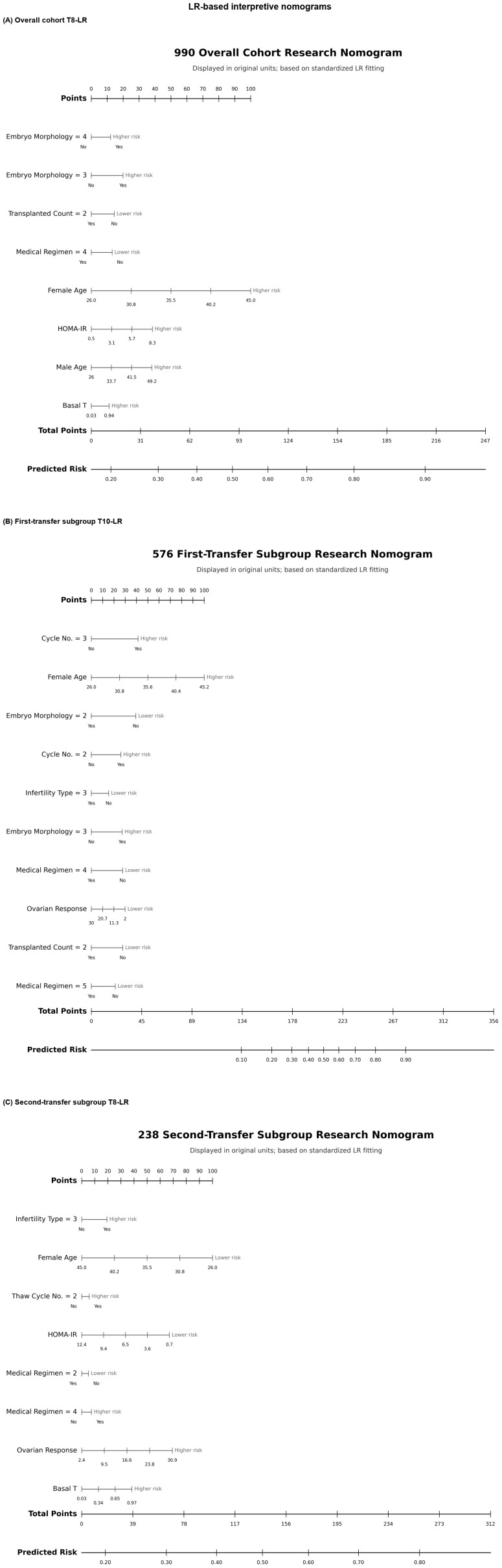
Interpretive nomograms based on the cohort-specific parallel LR tool templates across the three cohorts. In the first-transfer panel, cycle no. is interpreted as oocyte retrieval cycle number; ovarian denotes total AFC. encoded infertility-type, embryo-morphology, and medical-regimen labels denote factor-coded categorical levels rather than ordinal severity scores.

Taken together, the three templates should not be interpreted as interchangeable reduced versions of one common model. Instead, they represent population-matched predictive structures with different dominant pathways: the 990 template captures an average mixed-population architecture, the 576 template represents a contracted ovarian-reserve-age-endocrine pathway, and the 238 template represents an expanded multi-axis pathway in which reserve, endocrine, metabolic, infertility-type, and repeated-transfer process information jointly determine model discrimination. Correspondingly, **Figure 5** should be read as the operational translation of the selected parallel LR tool templates rather than as a replacement for the best nonlinear predictive templates.

#### Rule structure and operational expression in the 990 overall cohort

In the 990 overall cohort, both the classical association rules and the model interpretation results point to a relatively stable age-centered axis. The classical rules suggest that higher female age co-occurs with higher male age, embryo morphology information, intermediate HOMA-IR, and basal endocrine features such as Basal T, indicating that the dominant co-occurrence pattern in the mixed cohort still develops around the age background. The parallel T8-LR tool model retains Female Age, Male Age, encoded Embryo Morphology categories 3 and 4, HOMA-IR, Basal T, Medical Regimen category 4, and Number of embryos transferred = 2, indicating that the linear scoring structure is not driven by a single variable but by a joint age-embryo-metabolic-endocrine pattern.

At the predictive level, the best nonlinear model for the overall cohort is CatBoost with the T6 feature set, whereas T8-LR is retained as the parallel LR tool model rather than the performance-leading model. Its main value lies in translating the average risk structure of the mixed cohort into a cumulative scoring framework. Because the overall cohort covers different transfer histories and a broader clinical path, its rule structure is more diffuse than that of the subgroup analyses. Accordingly, the overall nomogram should be understood primarily as an operational summary of average risk organization in the full cohort.

#### Rule structure and operational expression in the 576 first-transfer subgroup

In the 576 first-transfer subgroup, the rule structure becomes markedly more concentrated than in the 990 overall cohort. The classical association rules indicate that lower AMH and higher Female Age form the dominant axis of the subgroup, which is further reinforced by basal hormone conditions such as Basal FSH and Basal PRL. Compared with the overall cohort, embryo-structure and treatment-structure information becomes less dominant, whereas the ovarian reserve-age-basal hormone axis becomes more prominent.

This interpretation is strengthened by the model results. The best predictive model in this subgroup is CatBoost with the T6 feature set, achieving higher discrimination than the overall cohort. However, because the best nonlinear model is not directly suited to nomogram translation, the study retains T10-LR as the parallel LR tool model. Because thaw cycle number is fixed at 1 in this subgroup, the retained Cycle No. terms are most appropriately interpreted as oocyte retrieval cycle numbers. The corresponding interpretive nomogram therefore keeps Oocyte Retrieval Cycle No. = 2, Female Age, Embryo Morphology = 2 (AX-like morphology group), Oocyte Retrieval Cycle No. = 1, Infertility Type = 3 (primary infertility), Embryo Morphology = 3 (compacting/cleavage-stage/rare morphology group), Medical Regimen = 4 (ovarian-stimulation cycle), Total AFC, Number of embryos transferred = 2, and Medical Regimen = 5 (hormone-replacement-therapy cycle). Therefore, the first-transfer nomogram functions as an operational scoring tool within a comparatively homogeneous subgroup while remaining linked to the main biological axis identified by the best predictive model.

#### Rule structure and operational expression in the 238 second-transfer subgroup

In the 238 second-transfer subgroup, both the main predictive model and the supplementary interpretation results indicate a further redistribution of the signal toward ovarian reserve, basal hormones, and transfer-course information. The best predictive model, CatBoost with the T10 feature set, prioritizes AMH, Basal P, primary infertility (Infertility Type = 3), Female Age, and Basal FSH, while the extended layer also incorporates Interval Days, BMI, Male Age, Basal PRL, and Basal LH. This pattern suggests that outcome variation in the second-transfer subgroup is shaped not only by ovarian reserve and age but also by infertility type, endocrine background, and repeated-transfer process information.

For operational translation, the study retains T8-LR as the parallel LR tool model in the second-transfer subgroup. This tool model emphasizes Infertility Type = 3 (primary infertility), Female Age, Thaw Cycle No. = 2, HOMA-IR, Medical Regimen = 2 (natural cycle), Medical Regimen = 4 (ovarian-stimulation cycle), Total AFC, and Basal T. Here, Thaw Cycle No. = 2 should be interpreted as a retained thaw-cycle category in the LR operational layer rather than as a universal constant that must hold for every second-transfer case. Compared with the full cohort, the second-transfer subgroup shows stronger local concentration of rules and a clearer internal stratification structure. Therefore, its interpretive nomogram is more appropriate for subgroup-specific risk stratification and mechanism comparison than for direct extrapolation to the overall population.

#### Comparative synthesis of the three cohorts and integration of interpretive nomograms

Taken together, the three cohorts show that Female Age and ovarian-reserve-related information recur throughout the analysis, but their structural roles are not identical across clinical contexts. The 990 overall cohort emphasizes a joint pattern composed of age background, embryo morphology, and metabolic-endocrine reinforcement. The 576 first-transfer subgroup is more strongly organized around AMH, Female Age, Basal FSH, and Basal PRL, indicating a more homogeneous reserve-hormone axis. The 238 second-transfer subgroup retains age and ovarian reserve while further strengthening the roles of Infertility Type, Basal P, Total AFC, HOMA-IR, and repeated-transfer information. Thus, subgroup heterogeneity is not reflected merely by sample size differences but by redistribution of interaction structure and dominant rule axes.

At the level of operational expression, the three interpretive nomograms shown in **Figure 5** should be interpreted as visual translations of the cohort-specific parallel LR tool templates rather than as substitutes for the best nonlinear predictive templates. Accordingly, exact feature identity between the nomogram and the best nonlinear model is neither required nor expected; the purpose of the nomogram layer is to convert the dominant subgroup-specific risk structure into an interpretable bedside scoring format. In **Figure 5**, Ovarian denotes Total AFC, and in the first-transfer panel Cycle No. should be interpreted as oocyte retrieval cycle number. The overall cohort nomogram summarizes the average risk organization of a mixed population. The first-transfer nomogram is the closest to the main biological axis of the best predictive model and therefore functions as the principal operational display in the manuscript. The second-transfer nomogram is more suitable for internal risk stratification and mechanism comparison within that subgroup. A concise cross-cohort summary of rule axes and interpretive positioning is provided in **Supplementary Table 3**.

## Discussion

The principal finding of this study is that transfer-order heterogeneity materially affects both predictive performance and explanation structure in live-birth modeling after freeze-all FET cycles. A single pooled overall-cohort model captures only the average risk organization of a mixed population, whereas subgroup-specific modeling identifies more coherent predictor combinations and more clinically usable interpretation pathways. In the final comparison, CatBoost + T6 was retained for the 990 and 576 cohorts, whereas CatBoost + T10 was retained for the 238 cohort.

Compared with existing prediction models for freeze-all or FET outcomes, the present study shows both consistency and extension. Consistent with previous freeze-all studies, female age and ovarian-reserve- or embryo-related variables remained important contributors (6–8). Broader FET prediction studies have also reported the importance of age, body mass index, embryo type or number, endometrial-related factors, and infertility type (14–17). The main difference is that earlier studies generally developed a single pooled model or focused on clinical pregnancy rather than live birth, whereas the present study explicitly treated transfer order as a modeling dimension. The resulting subgroup-specific configurations, namely a 6-feature template for the first-transfer setting and a 10-feature template for the second retained transfer-record setting, suggest that prediction models should be matched to the target clinical population rather than applied uniformly across heterogeneous FET records. Another novelty is the integrated interpretability framework, which combines feature-template stability, SHAP-based contribution patterns, rule-oriented explanation, and LR-based interpretive nomograms.

The main methodological implication is therefore not algorithm ranking alone, but model-population matching. Formal performance comparison was based on stratified 10-fold cross-validation, and binary metrics were summarized at ROC-derived Youden thresholds rather than at a fixed threshold of 0.5. At the same time, the study deliberately separates the best nonlinear predictive model from the parallel LR tool model: the former maximizes discrimination, whereas the latter provides an interpretable operational layer for nomogram-based bedside use. This type of separation is compatible with interpretable subgroup-learning frameworks that distinguish prediction from subgroup explanation (18). Development-stage subgroup-aware models should still be followed by later external validation when they are moved across settings (19).

A common biological backbone was retained across cohorts, centered on female age, ovarian-reserve-related information, and basal endocrine-metabolic markers. However, the way these signals were organized differed by transfer order. The 576 first-transfer subgroup showed a contracted structure in which AMH, Female Age, Basal FSH, Basal LH, HOMA-IR, and Basal PRL concentrated the dominant prognostic signal, indicating that first-transfer prediction is driven mainly by a reserve-age-basal-hormone axis. By contrast, the 238 second-transfer subgroup showed an expanded structure in which Basal P, Infertility Type, Interval Days, BMI, Male Age, and additional endocrine variables entered the retained template, indicating that repeated-transfer prediction depends on both biological background and accumulated treatment-course information.

This redistribution of features changes both the model discrimination pathway and the interpretation pathway. In the first-transfer subgroup, the main predictive task is to refine a relatively concentrated biological signal, so a compact nonlinear template can achieve strong discrimination while the LR tool model expands that same signal into an operational format using embryo morphology, cycle number, ovarian response, number of embryos transferred, and regimen-related variables. In the second-transfer subgroup, however, prediction cannot be reduced to a single dominant axis; the retained template must jointly capture endocrine context, infertility type, body-size-related background, and repeated-transfer process information. For this reason, exact feature identity between the best nonlinear model and the LR nomogram is not expected: the two layers are linked by subgroup-specific risk structure rather than by one-to-one variable duplication.

These results support a subgroup-aware prediction framework with five linked components: a common core domain assessed in all patients, subgroup-specific feature modules added according to transfer order, a best nonlinear model used when discrimination is the priority, a parallel LR tool model used when transparent scoring is needed, and a nomogram layer used for bedside explanation. This orientation is consistent with the PATH statement on predictive approaches to heterogeneity (20). It is also aligned with personalized evidence-based medicine frameworks that emphasize predictive heterogeneity (21). Related work on modeling and interpreting patient subgroups likewise supports structuring explanation around clinically meaningful strata (22). Ensemble-based subpopulation modeling further shows that one-model-for-all and group-specific strategies can be bridged rather than treated as mutually exclusive (23).

Several limitations should be considered. First, the study was conducted at a single center and did not include external validation; therefore, model transportability to other reproductive centers, laboratory protocols, embryo-assessment systems, and patient populations remains uncertain. Second, the 238-cycle second-transfer subgroup was relatively small, which may reduce model stability and increase uncertainty in subgroup-specific feature rankings. Third, because this was a prediction-model development study rather than a causal comparative study, propensity score matching was not performed; baseline comparisons were descriptive and should not be interpreted as balanced treatment-group contrasts. Fourth, although no patient contributed more than one record within any single modeling cohort, the overall cohort and transfer-order subgroups were related cohorts derived from the same source database, so cross-cohort differences should be interpreted as descriptive evidence of transfer-order-related heterogeneity. Finally, calibration assessment was based on the saved final test-split predictions rather than originally stored 10-fold out-of-fold probabilities, and prospective external validation is needed before clinical deployment.

Future work should prioritize multicenter external validation and model updating across centers and treatment strategies. Future studies should also incorporate richer representations of embryo morphology and broader predictor domains that include additional endometrial, hormonal, behavioral, and longitudinal treatment variables. Dedicated models for later repeated-transfer populations are also needed so that transfer-order heterogeneity can be characterized more continuously than in the present second-transfer comparison.

## Conclusion

In conclusion, overall-cohort modeling alone is insufficient for this prediction problem. First-transfer and second-transfer populations should be treated as clinically meaningful subgroups rather than absorbed into a pooled mixed cohort. Transfer-order subgroup modeling improves model-population matching and supports a clinically usable, trustworthy, and interpretable pre-transfer assessment scheme for live-birth probability.

## Data Availability

The dataset is not publicly available because it contains potentially identifiable clinical information from human participants. Access to the data is restricted by institutional data protection policies and ethics approval requirements. The dataset may be made available from the corresponding author upon reasonable request and subject to approval by the relevant institution and ethics committee. Requests to access these datasets should be directed to MT, 2432236136@qq.com.
