# Supplementary material for: Interpretable machine learning prediction of live birth after freeze-all FET cycles across transfer-order subgroups
Source: Front Endocrinol (Lausanne). 2026 Jul 17;17:1868575. doi: 10.3389/fendo.2026.1868575 (PMC13423638; doi:10.3389/fendo.2026.1868575)
Supplement: Supplementary file 1 [file Supplementaryfile1.docx]

**Supplementary Material**

**Supplementary Data**

This supplementary file was prepared using the Frontiers Supplementary Material template. It contains editable supplementary tables and one supplementary calibration figure that are directly relevant to the manuscript. Raw patient-level data files, serialized model objects, IDE configuration files, cache files, and intermediate image files were not included because they are not suitable for direct submission as supplementary Word material.

Supplementary material should be uploaded separately on submission. The captions are included in this file, and all supplementary tables are provided in editable Word format.

# Supplementary Figures and Tables

The following supplementary tables and figure provide baseline categorical characteristics, model-performance summaries, robustness analyses, calibration assessment, variable mapping, cross-cohort interpretive positioning, and rule-based interpretation results supporting the main manuscript.

**Supplementary Table S1. Selected baseline categorical characteristics across the three analytical cohorts.**

Values are presented as n (%). Between-cohort comparisons were performed descriptively using chi-square tests where applicable. Detailed encoded-variable meanings are provided in Supplementary Table S6.

| **Variable** | **990 overall cohort (n=990)** | **576 first-transfer subgroup (n=576)** | **238 second-transfer subgroup (n=238)** | **P value** |
| --- | --- | --- | --- | --- |
| Infertility type: Other | 24 (2.4%) | 18 (3.1%) | 4 (1.7%) | 0.817 |
| Infertility type: Primary infertility | 577 (58.5%) | 333 (58.0%) | 140 (59.3%) | 0.817 |
| Infertility type: Secondary infertility | 386 (39.1%) | 223 (38.9%) | 92 (39.0%) | 0.817 |
| Medical regimen: GnRHa + HRT cycle | 77 (7.8%) | 45 (7.8%) | 28 (11.8%) | <0.001 |
| Medical regimen: Natural cycle | 46 (4.6%) | 28 (4.9%) | 25 (10.5%) | <0.001 |
| Medical regimen: Ovulation-induction cycle | 6 (0.6%) | 3 (0.5%) | 5 (2.1%) | <0.001 |
| Medical regimen: Ovarian-stimulation cycle | 135 (13.6%) | 87 (15.1%) | 46 (19.3%) | <0.001 |
| Medical regimen: Hormone-replacement-therapy cycle | 726 (73.3%) | 413 (71.7%) | 134 (56.3%) | <0.001 |
| No. of embryos transferred: 1 | 562 (56.8%) | 310 (53.8%) | 95 (39.9%) | <0.001 |
| No. of embryos transferred: 2 | 428 (43.2%) | 266 (46.2%) | 143 (60.1%) | <0.001 |
| Embryo transferred day: 3 | 9 (0.9%) | 3 (0.5%) | 1 (0.4%) | <0.001 |
| Embryo transferred day: 4 | 435 (43.9%) | 264 (45.8%) | 111 (46.6%) | <0.001 |
| Embryo transferred day: 4+5 | 3 (0.3%) | 3 (0.5%) | 3 (1.3%) | <0.001 |
| Embryo transferred day: 4+6 | 1 (0.1%) | 1 (0.2%) | 0 (0.0%) | <0.001 |
| Embryo transferred day: 5 | 459 (46.4%) | 261 (45.3%) | 93 (39.1%) | <0.001 |
| Embryo transferred day: 5+6 | 6 (0.6%) | 6 (1.0%) | 11 (4.6%) | <0.001 |
| Embryo transferred day: 6 | 77 (7.8%) | 38 (6.6%) | 18 (7.6%) | <0.001 |
| Embryo transferred day: 6+7 | 0 (0.0%) | 0 (0.0%) | 1 (0.4%) | <0.001 |

**Supplementary Table S2. Top five model configurations ranked by ROC-AUC in each analytical cohort. Retained features are abbreviated when the configuration used more than six predictors.**

| **Cohort** | **Rank** | **Learner** | **Feature-selection template** | **No. features** | **AUC** | **Accuracy** | **F1-score** | **Retained features** |
| --- | --- | --- | --- | --- | --- | --- | --- | --- |
| 990 overall cohort | 1 | CatBoost | rf-shap-9999-T6 | 6 | 0.704 | 0.678 | 0.691 | AMH, Basal LH, Male Age, Female Age, HOMA-IR, Basal FSH |
| 990 overall cohort | 2 | CatBoost | lgbm-shap-9999-T8 | 8 | 0.698 | 0.672 | 0.678 | AMH, Basal LH, HOMA-IR, Female Age, Basal PRL, Basal FSH, ... |
| 990 overall cohort | 3 | Logistic regression | lasso-gini-0.005-T8 | 8 | 0.680 | 0.649 | 0.640 | Embryo Morphology = 4, Embryo Morphology = 3, Number of embryos transferred = 2, Medical Regimen = 4, Female Age, HOMA-IR, ... |
| 990 overall cohort | 4 | Logistic regression | lasso-gini-0.001-T6 | 6 | 0.674 | 0.635 | 0.608 | Oocyte Retrieval Cycle No. = 3, Embryo Morphology = 3, Number of embryos transferred = 2, Embryo Morphology = 4, Female Age, Medical Regimen = 4 |
| 990 overall cohort | 5 | Logistic regression | lasso-gini-0.025-T9 | 9 | 0.665 | 0.660 | 0.645 | Oocyte Retrieval Cycle No. = 2, Oocyte Retrieval Cycle No. = 3, Medical Regimen = 2, Female Age, Male Age, Medical Regimen = 3, ... |
| 576 first-transfer subgroup | 1 | CatBoost | lgbm-shap-9999-T6 | 6 | 0.812 | 0.750 | 0.657 | AMH, Basal FSH, Female Age, Basal LH, HOMA-IR, Basal PRL |
| 576 first-transfer subgroup | 2 | CatBoost | rf-gini-9999-T6 | 6 | 0.810 | 0.773 | 0.647 | AMH, Basal FSH, Male Age, Basal PRL, HOMA-IR, Female Age |
| 576 first-transfer subgroup | 3 | Random forest | lgbm-shap-9999-T6 | 6 | 0.794 | 0.740 | 0.632 | AMH, Basal FSH, Female Age, Basal LH, HOMA-IR, Basal PRL |
| 576 first-transfer subgroup | 4 | Logistic regression | lasso-gini-0.005-T10 | 10 | 0.770 | 0.770 | 0.650 | Oocyte Retrieval Cycle No. = 3, Female Age, Embryo Morphology = 2, Oocyte Retrieval Cycle No. = 2, Infertility Type = 3, Embryo Morphology = 3, ... |
| 576 first-transfer subgroup | 5 | Logistic regression | lasso-gini-0.001-T10 | 10 | 0.767 | 0.792 | 0.618 | Oocyte Retrieval Cycle No. = 2, Oocyte Retrieval Cycle No. = 3, Embryo Morphology = 3, Number of embryos transferred = 2, Medical Regimen = 5, Medical Regimen = 4, ... |
| 238 second-transfer subgroup | 1 | CatBoost | lgbm-shap-9999-T10 | 10 | 0.825 | 0.811 | 0.825 | AMH, Basal P, Infertility Type = 3, Female Age, Basal FSH, Interval Days, ... |
| 238 second-transfer subgroup | 2 | LightGBM | rf-shap-9999-T9 | 9 | 0.804 | 0.786 | 0.796 | AMH, Female Age, Infertility Type = 3, Total AFC, Basal FSH, Basal LH, ... |
| 238 second-transfer subgroup | 3 | LightGBM | lgbm-shap-9999-T4 | 4 | 0.803 | 0.778 | 0.807 | AMH, Basal P, Infertility Type = 3, Female Age |
| 238 second-transfer subgroup | 4 | XGBoost | lgbm-shap-9999-T4 | 4 | 0.796 | 0.786 | 0.797 | AMH, Basal P, Infertility Type = 3, Female Age |
| 238 second-transfer subgroup | 5 | Random forest | lgbm-shap-9999-T5 | 5 | 0.791 | 0.778 | 0.793 | AMH, Basal P, Infertility Type = 3, Female Age, Basal FSH |

**Supplementary Table S3. Cross-validated robustness summary of the top five optimized configurations in each cohort. Values are mean +/- standard deviation across stability runs.**

| **Cohort** | **Rank** | **Feature-selection template** | **Learner** | **No. features** | **AUC** | **Accuracy** | **F1-score** |
| --- | --- | --- | --- | --- | --- | --- | --- |
| 990 overall cohort | 1 | rf-shap-9999-T6 | CatBoost | 6 | 0.704 +/- 0.035 | 0.678 +/- 0.029 | 0.691 +/- 0.046 |
| 990 overall cohort | 2 | lgbm-shap-9999-T8 | CatBoost | 8 | 0.698 +/- 0.025 | 0.672 +/- 0.029 | 0.678 +/- 0.040 |
| 990 overall cohort | 3 | lgbm-shap-9999-T6 | CatBoost | 6 | 0.697 +/- 0.022 | 0.671 +/- 0.025 | 0.685 +/- 0.051 |
| 990 overall cohort | 4 | rf-shap-9999-T7 | CatBoost | 7 | 0.696 +/- 0.032 | 0.681 +/- 0.028 | 0.697 +/- 0.044 |
| 990 overall cohort | 5 | lgbm-shap-9999-T9 | CatBoost | 9 | 0.695 +/- 0.027 | 0.669 +/- 0.017 | 0.678 +/- 0.047 |
| 576 first-transfer subgroup | 1 | lgbm-shap-9999-T6 | CatBoost | 6 | 0.812 +/- 0.060 | 0.750 +/- 0.069 | 0.657 +/- 0.056 |
| 576 first-transfer subgroup | 2 | rf-gini-9999-T6 | CatBoost | 6 | 0.810 +/- 0.066 | 0.773 +/- 0.047 | 0.647 +/- 0.067 |
| 576 first-transfer subgroup | 3 | lgbm-shap-9999-T5 | CatBoost | 5 | 0.804 +/- 0.054 | 0.774 +/- 0.049 | 0.652 +/- 0.055 |
| 576 first-transfer subgroup | 4 | lgbm-shap-9999-T8 | CatBoost | 8 | 0.803 +/- 0.079 | 0.794 +/- 0.067 | 0.661 +/- 0.079 |
| 576 first-transfer subgroup | 5 | lgbm-shap-9999-T3 | CatBoost | 3 | 0.802 +/- 0.058 | 0.764 +/- 0.073 | 0.645 +/- 0.060 |
| 238 second-transfer subgroup | 1 | lgbm-shap-9999-T10 | CatBoost | 10 | 0.825 +/- 0.070 | 0.811 +/- 0.050 | 0.825 +/- 0.052 |
| 238 second-transfer subgroup | 2 | lgbm-shap-9999-T5 | CatBoost | 5 | 0.823 +/- 0.074 | 0.807 +/- 0.052 | 0.820 +/- 0.057 |
| 238 second-transfer subgroup | 3 | lgbm-shap-9999-T8 | CatBoost | 8 | 0.823 +/- 0.052 | 0.816 +/- 0.064 | 0.822 +/- 0.080 |
| 238 second-transfer subgroup | 4 | lgbm-shap-9999-T4 | CatBoost | 4 | 0.822 +/- 0.086 | 0.816 +/- 0.067 | 0.824 +/- 0.075 |
| 238 second-transfer subgroup | 5 | lgbm-shap-9999-T7 | CatBoost | 7 | 0.815 +/- 0.062 | 0.807 +/- 0.064 | 0.817 +/- 0.076 |

**Supplementary Table S4. Final test-split calibration metrics of the final retained best predictive models.**

Calibration metrics were calculated from saved final test-split predicted probabilities. Because out-of-fold predicted probabilities from all cross-validation folds were not stored in the available modeling artifacts, these metrics should be interpreted as supplementary final-split calibration evidence rather than as strict 10-fold out-of-fold calibration.

| **Cohort** | **Model** | **Final test-split n** | **Live births in final test split** | **Final-split AUC** | **Brier score** | **Log loss** | **Calibration intercept** | **Calibration slope** | **ECE (5 quantile bins)** |
| --- | --- | --- | --- | --- | --- | --- | --- | --- | --- |
| 990 overall cohort | CatBoost + T6 | 99 | 57 | 0.724 | 0.207 | 0.595 | -0.314 | 1.816 | 0.129 |
| 576 first-transfer subgroup | CatBoost + T6 | 58 | 16 | 0.818 | 0.156 | 0.543 | -0.010 | 0.544 | 0.081 |
| 238 second-transfer subgroup | CatBoost + T10 | 24 | 14 | 0.779 | 0.196 | 0.575 | -0.653 | 2.066 | 0.106 |

**Supplementary Figure S1. Final test-split calibration plots of the final retained best predictive models.**

The calibration plots were generated from saved final test-split predicted probabilities for the final retained best predictive models. Points summarize quantile-bin calibration patterns in each analytical cohort.


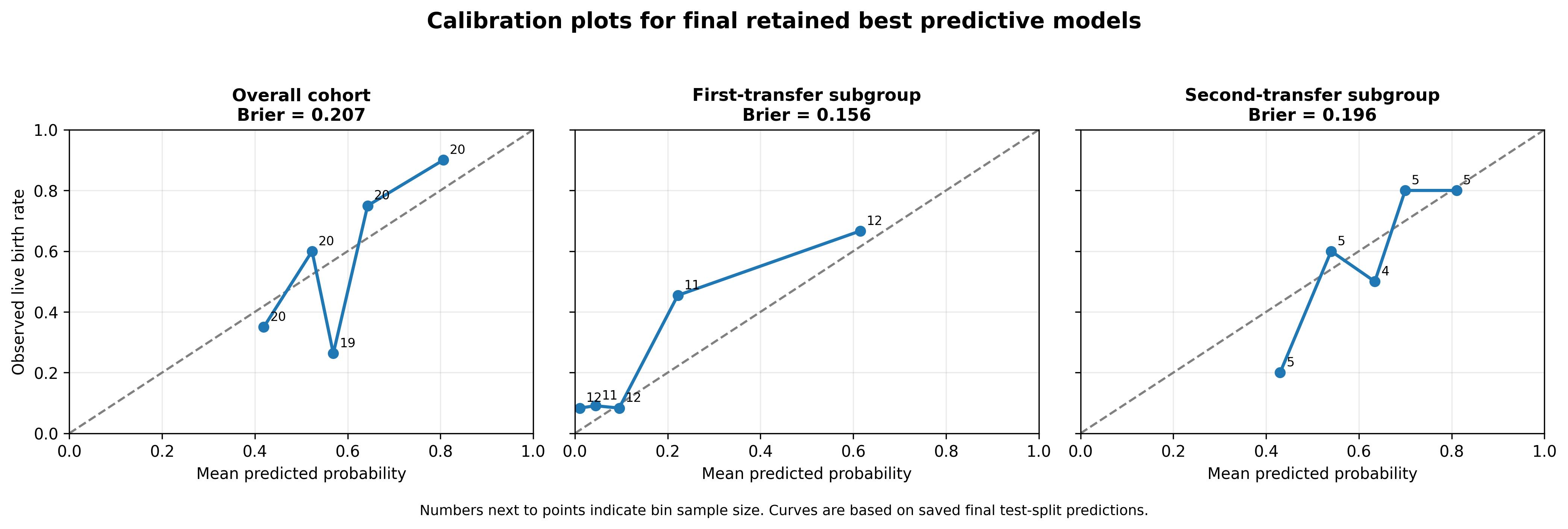


**Supplementary Table S5. Additional threshold-optimized model metrics for the final retained best predictive models and parallel LR tool models.**

Binary metrics were calculated at ROC-derived Youden thresholds. Specificity is not included in this table to maintain consistency with the main manuscript.

| **Cohort** | **Model role** | **Model** | **Feature template** | **No. of features** | **AUC** | **Accuracy** | **Sensitivity/Recall** | **Precision** | **F1** | **Optimal threshold** |
| --- | --- | --- | --- | --- | --- | --- | --- | --- | --- | --- |
| 990 Overall Cohort | Best predictive model | CatBoost + T6 | rf-shap-9999-T6 | 6 | 0.704 | 0.678 | 0.637 | 0.775 | 0.691 | 0.633 |
| 990 Overall Cohort | Parallel LR tool model | LR + T8 | lasso-gini-0.005-T8 | 8 | 0.680 | 0.649 | 0.576 | 0.773 | 0.640 | 0.631 |
| 576 First-Transfer Subgroup | Best predictive model | CatBoost + T6 | lgbm-shap-9999-T6 | 6 | 0.812 | 0.750 | 0.867 | 0.545 | 0.657 | 0.109 |
| 576 First-Transfer Subgroup | Parallel LR tool model | LR + T10 | lasso-gini-0.005-T10 | 10 | 0.770 | 0.770 | 0.732 | 0.631 | 0.650 | 0.211 |
| 238 Second-Transfer Subgroup | Best predictive model | CatBoost + T10 | lgbm-shap-9999-T10 | 10 | 0.825 | 0.811 | 0.797 | 0.876 | 0.825 | 0.649 |
| 238 Second-Transfer Subgroup | Parallel LR tool model | LR + T8 | lasso-gini-0.005-T8 | 8 | 0.737 | 0.773 | 0.890 | 0.770 | 0.815 | 0.530 |

**Supplementary Table S6. Feature-name mapping and encoded-variable interpretation for key variables appearing in the final models, tool models, or rule summaries. Encoded categorical levels should be interpreted as factor-coded indicators rather than ordinal clinical grades.**

| **Source variable name** | **English display name** | **Meaning or note** | **Used in groups** |
| --- | --- | --- | --- |
| AMH | AMH | Anti-Mullerian hormone | 238 second-transfer subgroup; 576 first-transfer subgroup; 990 overall cohort |
| diff_days | Interval Days | Number of days between the first and second transfer dates | 238 second-transfer subgroup |
| homa_ir | HOMA-IR | Homeostatic model assessment of insulin resistance | 238 second-transfer subgroup; 990 overall cohort |
| ovarian | Total AFC | Total antral follicle count, calculated as the sum of follicles in both ovaries | 238 second-transfer subgroup; 576 first-transfer subgroup; 990 overall cohort |
| 不孕类型_3 | Infertility Type = 3 | Primary infertility; factor-coded categorical indicator | 238 second-transfer subgroup; 576 first-transfer subgroup |
| 体重指数 | BMI | Body mass index | 238 second-transfer subgroup; 990 overall cohort |
| 基础FSH | Basal FSH | Basal follicle-stimulating hormone | 238 second-transfer subgroup; 576 first-transfer subgroup; 990 overall cohort |
| 基础LH | Basal LH | Basal luteinizing hormone | 238 second-transfer subgroup; 990 overall cohort |
| 基础P | Basal P | Basal progesterone | 238 second-transfer subgroup |
| 基础PRL | Basal PRL | Basal prolactin | 238 second-transfer subgroup; 576 first-transfer subgroup |
| 基础T | Basal T | Basal testosterone | 238 second-transfer subgroup; 990 overall cohort |
| 女方年龄 | Female Age | Female age | 238 second-transfer subgroup; 576 first-transfer subgroup; 990 overall cohort |
| 男方年龄 | Male Age | Male age | 238 second-transfer subgroup; 990 overall cohort |
| 移植胚胎形态_2 | Embryo Morphology = 2 | AX-like morphology group; factor-coded categorical indicator | 576 first-transfer subgroup |
| 移植胚胎形态_3 | Embryo Morphology = 3 | Compacting, cleavage-stage, or rare morphology group; factor-coded categorical indicator | 576 first-transfer subgroup; 990 overall cohort |
| 移植胚胎形态_4 | Embryo Morphology = 4 | Non-AA/AX blastocyst morphology group; factor-coded categorical indicator | 990 overall cohort |
| 用药方案类型_2 | Medical Regimen = 2 | Natural cycle; factor-coded categorical indicator | 238 second-transfer subgroup |
| 用药方案类型_4 | Medical Regimen = 4 | Ovarian-stimulation cycle; factor-coded categorical indicator | 238 second-transfer subgroup; 576 first-transfer subgroup; 990 overall cohort |
| 用药方案类型_5 | Medical Regimen = 5 | Hormone-replacement-therapy cycle; factor-coded categorical indicator | 576 first-transfer subgroup |
| 移植数_2 | Number of embryos transferred = 2 | Two embryos transferred; factor-coded categorical indicator | 576 first-transfer subgroup; 990 overall cohort |
| 第几解冻周期_2 | Thaw Cycle No. = 2 | Retained thaw-cycle category in the LR operational layer | 238 second-transfer subgroup |

**Supplementary Table S7. Cross-cohort interpretive positioning of the final predictive and parallel LR tool models.**

| **Cohort** | **Best predictive model** | **Parallel LR tool model** | **Dominant interpretive pathway** | **Main information domains** |
| --- | --- | --- | --- | --- |
| 990 overall cohort | CatBoost + T6 | T8-LR | Average mixed-population architecture | Age, ovarian reserve, male age, metabolic and basal endocrine markers |
| 576 first-transfer subgroup | CatBoost + T6 | T10-LR | Contracted ovarian-reserve-age-basal-hormone pathway | AMH, female age, basal FSH, basal LH, basal PRL, HOMA-IR |
| 238 second-transfer subgroup | CatBoost + T10 | T8-LR | Expanded multi-axis repeated-transfer pathway | Total AFC reserve, basal hormones, infertility type, interval days, BMI, male age |

**Supplementary Table S8. Rule-based interpretation results for selected model layers. Thresholds are shown on the processed modeling scale after preprocessing and standardization.**

| **Cohort** | **Model layer** | **Rank** | **Rule** | **Accuracy** | **Precision** | **Recall** | **F1-score** |
| --- | --- | --- | --- | --- | --- | --- | --- |
| 990 overall cohort | Parallel LR tool model | 1 | Female Age > -0.5204929411411285 and Male Age > -0.5064887851476669 | 0.788 | 0.951 | 0.763 | 0.847 |
| 990 overall cohort | Parallel LR tool model | 2 | Medical Regimen = 4 <= 0.5 and Female Age > -0.5204929411411285 and Male Age > -0.7102368175983429 | 0.778 | 0.966 | 0.737 | 0.836 |
| 990 overall cohort | Parallel LR tool model | 3 | Female Age > -0.28931841999292374 and Male Age > -0.30274076014757156 | 0.768 | 1.000 | 0.697 | 0.822 |
| 576 first-transfer subgroup | Parallel LR tool model | 1 | Female Age > 1.1747023463249207 and Embryo Morphology = 3 > 0.5 and Total AFC <= -0.1227651983499527 | 0.983 | 1.000 | 0.750 | 0.857 |
| 576 first-transfer subgroup | Parallel LR tool model | 2 | Female Age > 1.1747023463249207 and Infertility Type = 3 <= 0.5 and Embryo Morphology = 3 > 0.5 | 0.983 | 1.000 | 0.750 | 0.857 |
| 576 first-transfer subgroup | Parallel LR tool model | 3 | Female Age > 0.9432972371578217 and Embryo Morphology = 3 > 0.5 and Total AFC <= -0.9723638594150543 | 0.983 | 1.000 | 0.750 | 0.857 |
| 238 second-transfer subgroup | Best predictive model | 1 | AMH > -0.5177507102489471 and Basal P <= 4.905441403388977 and Male Age <= 1.1793299913406372 | 0.875 | 1.000 | 0.833 | 0.909 |
| 238 second-transfer subgroup | Best predictive model | 2 | AMH > -0.5177507102489471 and Basal P <= 5.20816445350647 and Female Age <= 1.148483157157898 | 0.875 | 1.000 | 0.833 | 0.909 |
| 238 second-transfer subgroup | Best predictive model | 3 | Female Age <= 1.148483157157898 and Basal FSH <= 1.2740300297737122 and Male Age <= 1.1793299913406372 | 0.833 | 0.889 | 0.889 | 0.889 |
| 238 second-transfer subgroup | Parallel LR tool model | 1 | HOMA-IR <= 1.1990611553192139 and Total AFC > -0.14018144458532333 | 0.792 | 0.938 | 0.789 | 0.857 |
| 238 second-transfer subgroup | Parallel LR tool model | 2 | Female Age <= 0.6929181516170502 and HOMA-IR <= 5.679049491882324 and Total AFC > -0.6326269805431366 | 0.750 | 0.933 | 0.737 | 0.824 |
| 238 second-transfer subgroup | Parallel LR tool model | 3 | Female Age <= 0.6929181516170502 and HOMA-IR <= 5.679049491882324 and Total AFC > -0.755738377571106 | 0.750 | 0.933 | 0.737 | 0.824 |
